# Sewing Machines

**Published:** 1867-05

**Authors:** 


*Sewing Machines.*—Dr. Down, of the Redhill asylum for Idiots, has recently read a paper upon the detrimental action of these machines upon the health of those who habitually use them. At his hospital in London, his patients who had been constantly employed in working sewing machines, suffered from pallor, lassitude, pain in the back and leucorrhea. Some of them became conscious of the injury they were receiving from observation of their own symptoms, and abandoned an occupation manifestly detrimental. These ill effects attend the use of the large, heavy machines worked with both feet. The lighter apparatus worked by a single treadle is not so injurious.

